# Expression of p16 Within Myenteric Neurons of the Aged Colon: A Potential Marker of Declining Function

**DOI:** 10.3389/fnins.2021.747067

**Published:** 2021-10-07

**Authors:** Alexandra Palmer, Sarah Epton, Ellie Crawley, Marilisa Straface, Luke Gammon, Meghan M. Edgar, Yichen Xu, Shezan Elahi, Joanne Chin-Aleong, Joanne E. Martin, Cleo L. Bishop, Charles H. Knowles, Gareth J. Sanger

**Affiliations:** ^1^Center for Neuroscience, Surgery and Trauma, Blizard Institute, Barts and The London School of Medicine and Dentistry, Queen Mary University of London, London, United Kingdom; ^2^Barts Health NHS Trust, Department of Colorectal Surgery and Pathology, The Royal London Hospital, London, United Kingdom; ^3^Center for Cell Biology and Cutaneous Research, Blizard Institute, Barts and The London School of Medicine and Dentistry, Queen Mary University of London, London, United Kingdom; ^4^Gastroenterology Drug Discovery Unit, Takeda Pharmaceuticals, San Diego, CA, United States; ^5^Center for Inflammation and Therapeutic Innovation Blizard Institute, Barts and The London School of Medicine and Dentistry, Queen Mary University of London, London, United Kingdom

**Keywords:** senescence, p16, aging, human, colon, myenteric neuron

## Abstract

Human colonic neuromuscular functions decline among the elderly. The aim was to explore the involvement of senescence. A preliminary PCR study looked for age-dependent differences in expression of *CDKN1A* (encoding the senescence-related p21 protein) and *CDKN2A* (encoding p16 and p14) in human ascending and descending colon (without mucosa) from 39 (approximately 50: 50 male: female) adult (aged 27–60 years) and elderly donors (70–89 years). Other genes from different aging pathways (e.g., inflammation, oxidative stress, autophagy) and cell-types (e.g., neurons, neuron axonal transport) were also examined. Unlike *CDKN1A, CDKN2A* (using primers for p16 and p14 but not when using p14-specific primers) was upregulated in both regions of colon. Compared with the number of genes appearing to upregulate in association with temporal age, more genes positively associated with increased *CDKN2A* expression (respectively, 16 and five of 44 genes studied for ascending and descending colon). Confirmation of increased expression of *CDKN2A* was sought by immunostaining for p16 in the myenteric plexus of colon from 52 patients, using a semi-automated software protocol. The results showed increased staining not within the glial cells (S100 stained), but in the cytoplasm of myenteric nerve cell bodies (MAP2 stained, with identified nucleus) of ascending, but not descending colon of the elderly, and not in the cell nucleus of either region or age group (5,710 neurons analyzed: *n* = 12–14 for each group). It was concluded that increased p16 staining within the cytoplasm of myenteric nerve cell bodies of elderly ascending (but not descending) colon, suggests a region-dependent, post-mitotic cellular senescence-like activity, perhaps involved with aging of enteric neurons within the colon.

## Introduction

Growing old is associated with an increased incidence of constipation, fecal impaction and incontinence, with reduced quality of life. These changes have been associated with age-related lifestyle changes, disease and use of medications which affect gastrointestinal (GI) functions (O’Mahony et al., 2002).

The effects of aging on intestinal functions have been widely studied in animals, especially rodents; many report reduced intestinal motility with loss of enteric neurons, although the latter remains controversial (e.g., Saffrey, 2004). However, compared with humans, rodents have high metabolic rates, high rates of aging and key differences in GI anatomy, neuronal functions, receptor pharmacology and molecular structures, compounded by genetic variation between different strains of laboratory rodents capable of influencing, for example, how advancing age affects GI functions (Wu et al., 2003; Keane et al., 2011; Sanger et al., 2011; Richardson et al., 2016). Accordingly, it is important to study the effects of aging on human GI functions. In healthy aged human intestine, major functions remain unchanged, including epithelial resistance, nerve-evoked secretion (Krueger et al., 2016), muscle tension developed during contraction, and numbers of myenteric neurons within different regions of the colon (Broad et al., 2019). Nevertheless, in many non-GI tissues, changes in several molecular pathways are associated with aging, including those involved with development of chronic senescence and inflammation (Bhatia-Dey et al., 2016; Rea et al., 2018). Further, some studies in human colon report loss of neuromuscular functions among the elderly. For example, increasing age (20–75 years) reduced the ability of distal colon muscle to stretch (Watters et al., 1985). In addition, cholinergic function was found to be reduced with increasing age in ascending colon, associated with increased immunostaining of myenteric cell bodies for choline acetyltransferase (ChAT). Since ChAT is synthesized within nerve cell bodies and transported to axon terminals for acetylcholine synthesis, these findings suggest an age-related decline in cholinergic axon transport (Broad et al., 2019). More recently, an age-related decline in mesenteric nerve nociception was found in human colon (Cibert-Goton et al., 2020). Finally, expression of mRNA for the senescence markers p16 and p21, and for elements of the senescence-associated secretory phenotype (SASP) were increased within mucosal biopsies from aged human colon, especially in individuals consuming normal western diets (argued to increase cellular senescence) and not under calorie restriction (Fontana et al., 2018).

p14, p16, and p21 are markers for cellular senescence. The p21 protein, encoded by the *CDKN1A* gene, is associated with acute senescence in many tissues in organisms of all age (Stein et al., 1999), including upregulation in myenteric neurons of the mouse intestine following short-term caloric restriction (Jurk et al., 2012). This involves exit of the cell from the cell cycle and secretion of inflammatory mediators, with affected cells usually rapidly cleared by the immune system. The expression of p21 may be facilitated by p14, encoded by the *CDKN2A* gene (Sherr, 2006; Bieging et al., 2014). However, with increasing age, cells can enter chronic senescence, involving chromatin remodeling and transcription changes (van Deursen, 2014), thought to be potentially causative for different aspects of aging (Bhatia-Dey et al., 2016). The p16 protein, also encoded by the *CDKN2A* gene, is a marker for chronic cellular senescence (Stein et al., 1999). p16 is a cell cycle regulator usually expressed in the nucleus, where it interacts with CDK4 to prevent the G1/S transition (Serrano et al., 1993; Ciesielska et al., 2017).

Aging of the human bowel is poorly understood, but a decline in enteric cholinergic function (Broad et al., 2019) and perhaps other functions, raises questions about the existence and roles of age-related degenerative pathways in the aging bowel. A senescent-like state has previously been noted in mature post-mitotic enteric neurons in the intestine of elderly mice (Jurk et al., 2012) but similar studies have not yet been conducted for the human enteric nervous system. Additionally, regional differences in the age-related decline of enteric cholinergic function (Broad et al., 2019) highlighted the importance of considering ascending and descending colon as separate regions. The current study began as a pilot investigation which used qPCR to explore the possibility that senescence might be upregulated in the mucosa-free wall of human ascending and descending/sigmoid colon (containing multiple cell-types including muscle and enteric neurons) of the “elderly” (≥70 years), compared with younger “adults” (25–60 years). Analysis of the data generated, highlighted a potential age-related increase in expression of the gene for p16, particularly in the ascending colon. The involvement of p16 with senescence, often considered as the exit of a proliferating cell from the cell cycle (Serrano et al., 1993; Ciesielska et al., 2017), created the hypothesis that potentially proliferative cells within the myenteric plexus had become senescent in the aged human colon. We focused on the enteric neurons (because a senescent-like state may exist in enteric neurons of the aged mouse; Jurk et al., 2012) and glial cells (because of their loss within the myenteric plexus of aged rats and potential to undergo neurogenesis in mice following damage to enteric ganglia; Phillips et al., 2004; Laranjeira et al., 2011). Immunofluorescence was used to confirm the change and localize the upregulation of the p16 protein. The results identified p16 within the cytoplasm of myenteric nerve cell bodies, not the nucleus, and not in glial cells, implying a role for post-mitotic cellular senescent-like mechanisms in declining enteric neuron functions during aging.

## Materials and Methods

### Human Tissues

Macroscopically-normal ascending and descending/sigmoid (referred to hereon as descending) colon was taken 5–10 cm from the tumor following surgery for non-obstructing bowel cancer, as described previously (Broad et al., 2019). No patient had previous chemoradiotherapy or diagnosis of inflammatory bowel disease. Tissues were immersed into Krebs solution (mmol L^–1^: NaCl 121.5, CaCl_2_ 2.5, KH_2_PO_4_ 1.2, KCl 4.7, MgSO_4_ 1.2, NaHCO_3_ 25, glucose 5.6 at room temperature, equilibrated with 5% CO_2_ and 95% O_2_) and transferred to the laboratory within 2 h of surgery. Here they were dissected for separate muscle and mucosa storage in RNAlater (Sigma Aldrich) and/or prepared for fixing into full thickness paraffin blocks.

### RNA Extraction

Extraction was from colon (mucosa removed) previously stored at −80°C in RNAlater. This was homogenized in TRIzol and RNA extraction carried out using the Direct-zol RNA Miniprep Kit (Zymo Research) according to manufacturer’s instructions.

### qPCR

cDNA synthesis was performed using the SuperScript VILO cDNA Synthesis Kit (Invitrogen). SYBR Green reagents (Thermo Fisher) were used for qPCR analysis, and primers were used at 40 μM concentration (Supplementary Table 4). mRNA concentrations were normalized against two commonly used “housekeeping” genes: GAPDH and ATP5B (determined by geNorm kit, PrimerDesign).

Tissue samples were grouped according to age and region of colon. Age groups were defined as “adults” (25–60 years) and “elderly” (≥70 years) and ascending colon and descending colon were examined separately; adult ascending *n* = 9, elderly ascending *n* = 10, adult descending *n* = 10, elderly descending *n* = 10. These groups were taken from previously published research which defined an age- and region-dependent decline in neuromuscular function of human colon (Broad et al., 2019). The adult samples had an age range of 27–60 years (median 53) and were 53% male. The elderly samples had an age range of 70–89 years (median 79) and were 50% male.

### CD45 Analysis

Using formalin-fixed, paraffin-embedded 4 μm sections, immunohistochemistry against CD45 (Roche, 760–4279) was carried out using the Ventana system. Slides were counterstained using haematoxylin and digitally scanned. Samples were analyzed (*n* = 10 for each of four groups; adult and elderly ascending colon, adult and elderly descending colon; a separate cohort to that used for qPCR and p16 immunofluorescence). Adult samples were 30–60 years of age (median 56) and 50% male. Elderly samples were 70–91 years of age (median 77.5) and 50% male. Each sample consisted of two, non-consecutive wax sections, of 4 μm thickness.

Images were taken at 20x magnification using automated setting for focus and using set exposures across all samples. These were analyzed by two independent observers blind to age and colon region. Up to 24 regions of interest (ROIs), 365 μm × 365 μm, were placed on each section using NDP View 2 and FIJI software’s. To meet inclusion criteria, at least four ROIs were analyzed in each region of muscle: 4–8 in longitudinal muscle, 4–8 in circular muscle adjacent to longitudinal muscle, 4–8 in circular muscle adjacent to mucosa. Therefore, for each patient up to 48 ROIs were analyzed, with an area of 6.3 mm^2^. Images were judged by whether enough ROIs could be taken to meet inclusion criteria. Final sample sizes for adult and elderly ascending colon, adult and elderly descending colon were *n* = 7, 9, 10 and 9, respectively, for observer 1, *n* = 6, 6, 9 and 8 for observer 2. Together, a total of 340 mm^2^ was analyzed (range 3.2–6.3 mm^2^/sample; median 5.9 mm^2^).

For each ROI, CD45-positive cells were counted, and FIJI software used to estimate total cell count from the haematoxylin-stained nuclei.

### p16 Analysis

4 μm sections were dewaxed in xylene, rehydrated in ethanol and distilled water, unmasked using citrate buffer (pH 6), and blocked in 1% BSA. Primary antibodies were against p16^INK4A^ (1:200, ProteinTech, 10883-1-AP, AB_2078303), S100 (1:3,000; Abcam, ab14849, AB_301508) and MAP2 (1:500; Abcam, ab5392, AB_2138153). Secondary antibodies [Abcam, ab150073 (AB_2636877), ab150105 (AB_2732856), ab175477] were applied at a concentration of 1:500, with DAPI and HCS CellMask^TM^ (1 μg/ml and 1:200,000 respectively, both Thermo Scientific).

A p16^INK4A^ fusion protein (ProteinTech; Ag1328) was used as blocking peptide for the p16 antibody. A volume was used equivalent to 5x the amount of antibody by weight. During control experiments antibody solutions were incubated with or without blocking peptide for 1 h at room temperature prior to application (Supplementary Figure 2).

Myenteric plexus was imaged using IN Cell Analyser 2200 and analyzed using IN Cell Developer Toolbox software (both GE Healthcare). Images were taken at 20x magnification using automated setting for focus and exposure. DAPI and MAP2 staining of myenteric neurons were clearly defined and showed distinct nuclei/cells. To define regions of positive staining, thresholds were systematically set relative to the smooth muscle (negative internal control for staining within each section) (Figures 6A–C′). This process was performed whilst blinded to the donor age and region of colon. This enabled a robust, automated protocol for quantification of positively stained regions. Visual inspection was routinely performed to confirm the validity of the automated protocol.

**FIGURE 1:**
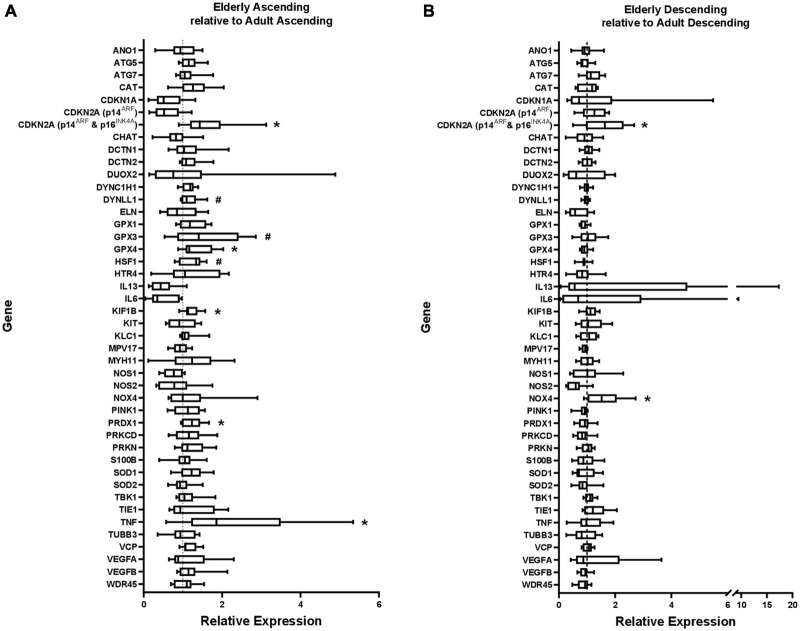
Gene expression in **(A)** elderly ascending colon and **(B)** elderly descending colon, shown relative to gene expression in adult ascending or descending colon, which has been normalized to 1 for each gene (shown by dashed line). ATP5B and GAPDH were used as endogenous controls. Results represent fold-change differences. #*p* < 0.1 and *p < 0.05. Student’s *t-*test used to calculate *P* value for each gene, comparing between age groups.

**FIGURE 2:**
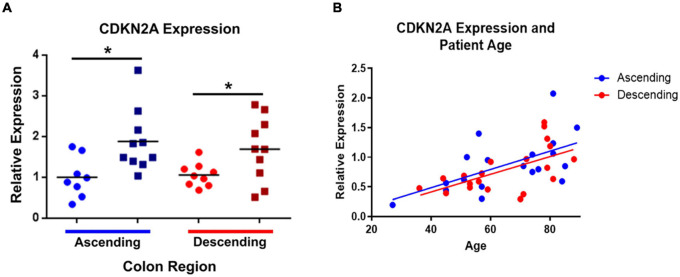
*CDKN2A* gene expression (using primers for p16^INK4A^ and p14^ARF^) is significantly increased in elderly ascending and descending colon. Panel **(A)** compares the elderly and adult samples from both regions, relative to expression in adult ascending colon, which has been normalized to 1. ATP5B and GAPDH were used as endogenous controls. Results represent fold-change differences. Adult samples (25–60 years old) are represented by •. Elderly samples (70 + years old) are represented by ■. Bars indicate data mean. *P < 0.05. Student’s *t-*test used to calculate *P* value for each gene, comparing between age groups. Panel **(B)** shows the correlations between *CDKN2A* gene expression and increasing age (years). *P* values for linear regression lines are, respectively, 0.0087 and 0.0063 for ascending and descending colon (calculated using Prism).

**FIGURE 3:**
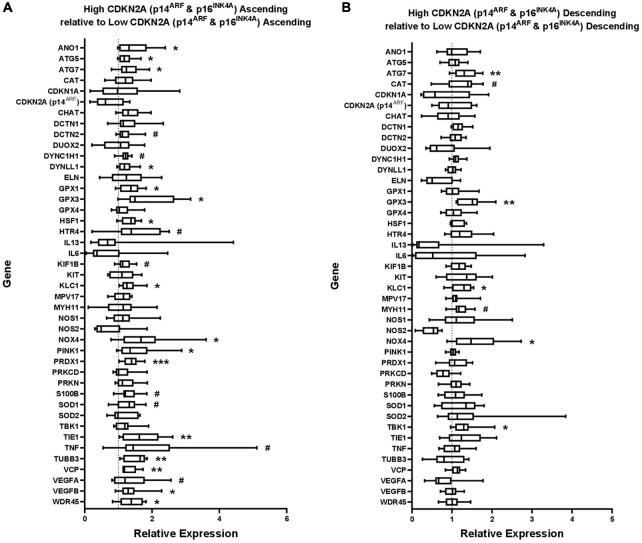
Gene expression in **(A)** ascending colon and **(B)** descending colon with high *CDKN2A* (p16^INK4A^ and p14^ARF^) expression, shown relative to gene expression in ascending or descending colon with low p16 expression, which has been normalized to 1 for each gene (shown by dashed line). ATP5B and GAPDH were used as endogenous controls. Results represent fold-change differences. #*P* < 0.1; *P < 0.05; **P < 0.01; and ***P < 0.001. Student’s *t-*test used to calculate *P* value for each gene, comparing between age groups.

**FIGURE 4:**
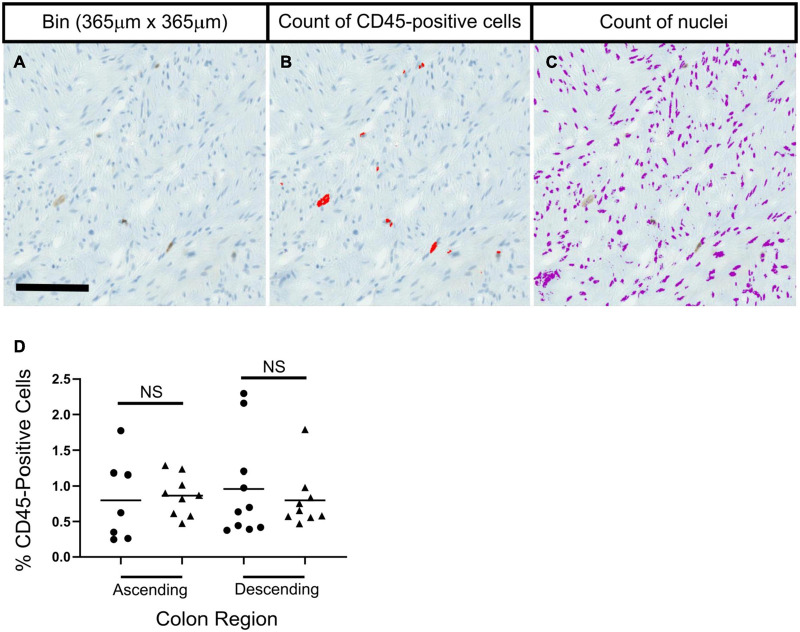
No significant age-related differences were seen in the percentage of CD45-positive cells in colon sections in ascending or descending colon. Panels **(A–C)** show methods used to calculate the percentage of CD45-positive cells. A bin for analysis was taken **(A)**, and thresholding was used to identify CD45-positive cells and nuclei, which were then counted. Statistical tests compared results for each colon region between age groups; adult (25–60 years old; •) and elderly (70 + years old; ▲). Bars indicate data mean. Data was collected separately for: longitudinal muscle, circular muscle near the myenteric plexus, and circular muscle near the mucosa. Data shown represents an average from the three regions from each individual. No statistical significance was found when comparing age groups for the three regions individually, or when grouped. Data was analyzed by two independent observers. Results from one observer are shown in panel **(D)** as an example.

**FIGURE 5:**
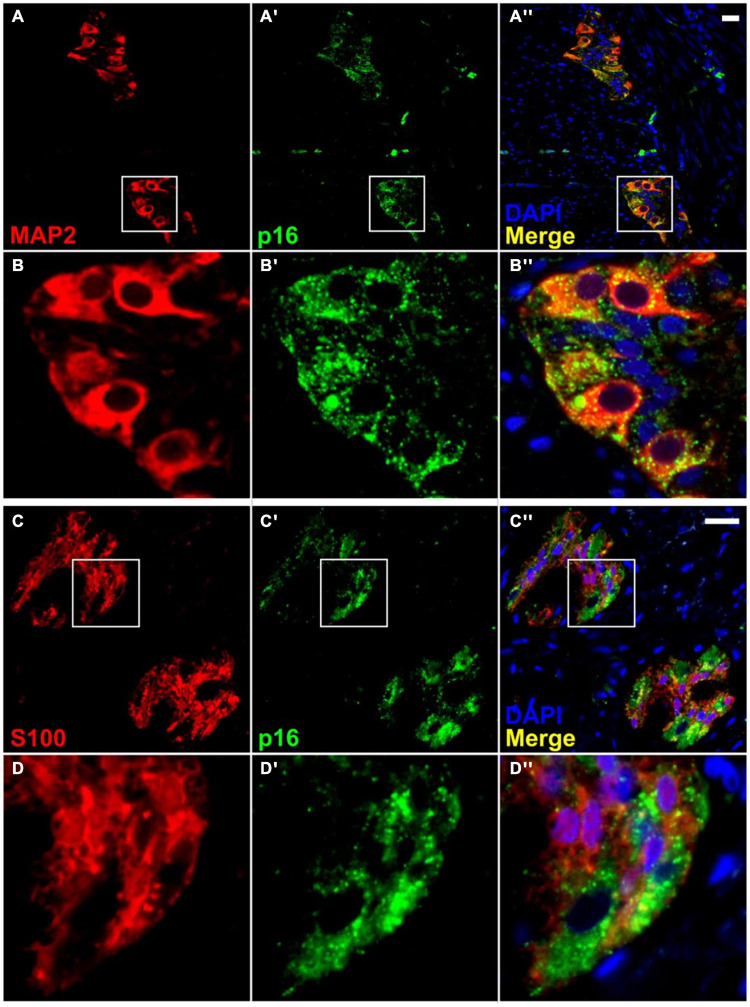
Co-localization of p16 with a neuronal marker in the myenteric plexus, but with little or no co-localization with a glial cell marker. Immunofluorescence for a neuronal marker, MAP2 **(A,B)**, shows co-staining with p16 (**A′,A″,B′,B″)**. However, immunofluorescence for a glial cell marker, S100 **(C,D)**, shows minimal co-localization with p16 **(C′,C″,D′,D″)**. It is noteworthy that p16 staining is present mainly in the neuronal cytoplasm and not the nucleus. Most nuclear staining observed was peripheral, and it was impossible to rule out overlap with the cytoplasm. Neuronal nuclei are present but do not take up DAPI stain as well as other cell types. S100 staining was not further quantified. Scale bar represents 25 μm.

**FIGURE 6:**
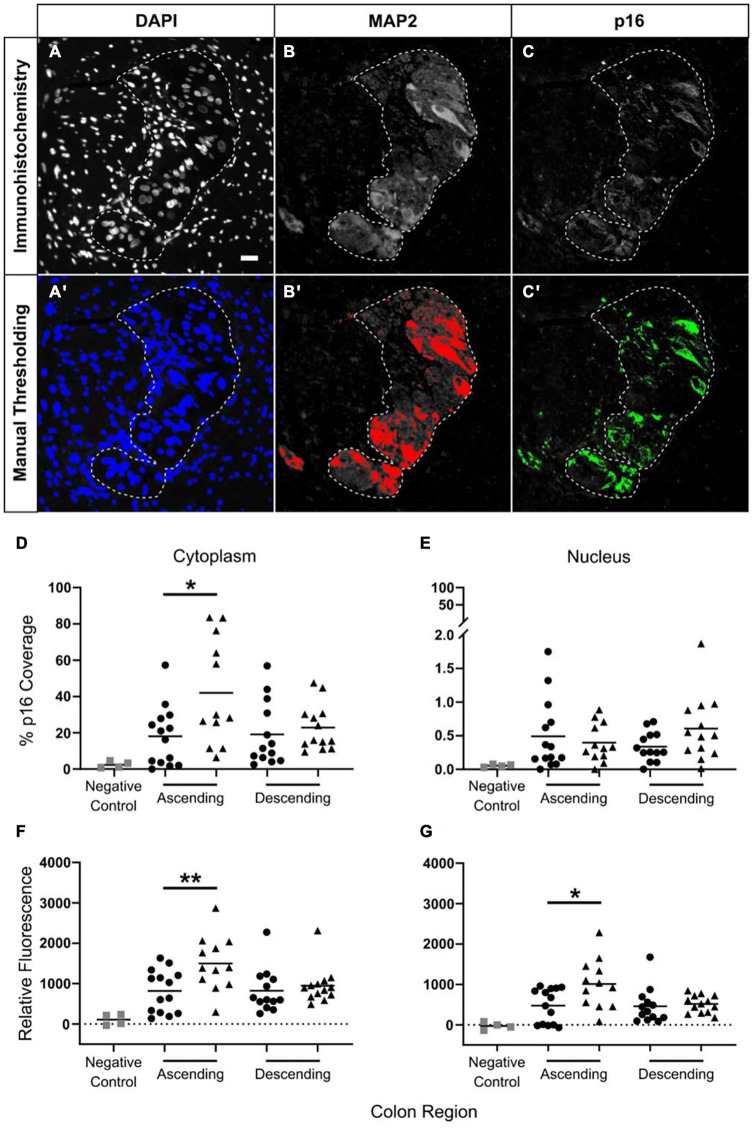
Increased p16 protein expression with advanced age in neuronal cytoplasm in ascending but not descending colon. Thresholding was used to identify positive immunostaining. Panels **(A–C)** show the original immunostaining, and panels **(A′,B′,C′)** show the respective thresholding, with areas of positive staining shown in color. Images DAPI **(A,A′)** and MAP2 **(B,B′)** determined positive and negative areas of staining. For p16 **(C,C′)** the level of fluorescence in the surrounding smooth muscle was measured, and the threshold was set at twice this background level. Scale bar in A is 25 μm. The results were calculated using two methods. The first, is a calculation of the percentage of area covered by p16-positive staining in the region of interest [shown in panels **(D,E)**] as determined by thresholding. The second method is a measurement of the average level of p16 fluorescence in the region of interest relative to background **(F,G)**. Results are for the cytoplasm **(D,F)** and the nucleus **(E,G)**. Bars indicate data mean. *N* = 52: adult ascending colon *n* = 14, elderly ascending *n* = 12, adult descending *n* = 13, elderly descending *n* = 13. *p < 0.05 and **p < 0.01. Student’s *t* test used to compare between age groups. Panel **(D)** shows a significant increase in p16 coverage in the neuronal cytoplasm of the ascending colon when comparing samples from adult (25–60 years old; •) and the elderly (70+ years old; ▲). Panel **(E)** shows no age-related changes in p16 coverage in neuronal nuclei for either colon region. Panels **(F,G)** show age-related increases in p16 fluorescence in the ascending colon in cytoplasm **(F)** and nuclei **(G)** of myenteric plexus neurons; changes in the nuclei may be due to overlap with the cytoplasm.

Only MAP2-positive cells associated with a nucleus were analyzed. p16 fluorescence was measured in the nucleus and cytoplasm (region of MAP2 staining minus the nucleus, as shown by DAPI) using two methods. The first involved automated quantification of the average fluorescence intensity within the ROI, from which background fluorescence of smooth muscle (the internal control) was subtracted. This detected changes in cell-wide, dispersed fluorescence, but may not have detected more localized changes in fluorescence, such as foci. The second method involved automated quantification of the proportion of the ROI positive for p16 fluorescence. In this instance, the threshold for measurement was systematically defined as twice the level of background fluorescence in smooth muscle. This detected changes in fluorescent foci, although it may not have detected dispersed changes in fluorescence in the ROI. To determine the proportion of MAP2-stained cells in which there was a high level of confidence for p16 staining we counted the number of cells in which 50% or more of the cytoplasmic area was above threshold for p16 staining.

In total, samples from 58 patients were analyzed, with 14–16/group (adult and elderly, ascending and descending colon). Samples which did not contain myenteric plexus or did not have clearly defined MAP2-positive cells were removed from analysis. Final analysis was carried out using samples from 52 individuals (adult ascending *n* = 14, elderly ascending *n* = 12, adult descending *n* = 13, elderly descending *n* = 13). Adult samples were 27–60 years of age (median 56) and 52% male. Elderly samples were 70–91 years of age (median 79) and 52% male. Four individuals were additionally used for negative controls without p16 antibody. Final analysis included 5,710 neurons, with a median of 71 (range 13–293) neurons/patient.

### Statistics and Data Analysis

Data from qPCR were analyzed using ExpressionSuite (Thermo Fisher Scientific, Walton, MA, United States) software using the ΔΔCt method and expressed as fold-changes comparative to the control group. The qPCR data and those from the immunohistochemistry/immunofluorescence experiments were analyzed using student’s *t*-tests to compare between age groups for each region of colon. Proprietary software (GraphPad Prism v8.0, San Diego, CA, United States) was used for all statistical tests. A *P*-value of ≤0.05 indicated statistical significance.

## Results

### Changes in Gene Expression Determined by qPCR

Analysis was performed on RNA extracted from full thickness colon, with mucosa removed, containing a mixture of cell types, including but not limited to neurons, glial cells, interstitial cells of Cajal and smooth muscle. Genes were chosen from the literature to represent senescence and other pathways reported to be altered with advancing age (oxidative stress, inflammation, angiogenesis, autophagy; Lahteenvuo and Rosenzweig, 2012; He et al., 2013; Bhatia-Dey et al., 2016; Franceschi et al., 2017; Liguori et al., 2018), pathways involved in neuron axonal transport (suggested to decline in enteric cholinergic neurons of ascending colon of the elderly; Broad et al., 2019) and genes to mark specific cell types, such as interstitial cells of Cajal (ICCs), neurons and neuronal subtypes. Supplementary Table 1 provides the full list of genes studied, with brief descriptions.

Within the ascending colon, significant age-related increases in expression occurred for five of the 44 genes studied, including *CDKN2A* and *TNF*. For *CDKN2A*, the p16 product was not examined separately (the primers were for p16^INK4A^ and p14^ARF^). The p14 product tended to increase but this was not statistically significant (Figure 1A and Supplementary Table 2). In the descending colon only two of the 44 genes were significantly upregulated in the elderly (Figure 1B and Supplementary Table 2), including *CDKN2A* (but not the p14 product of this gene), the only gene upregulated in an age-dependent manner in both regions (Figure 2). In both regions of colon, the increases were small but statistically significant, e.g., *TNF* increased 2.3-fold and *CDKN2A* increased 1.6-fold in ascending colon of the elderly compared to adult ascending colon (*P* < 0.05). A sub-analysis of gene expression for *CDKN2A, DUOX2, CDKN1A, TNF*α, *IL13* and *IL6* (exemplars showing relatively high variation in expression) failed to show significant sex-related differences in levels of expression (one way ANOVA with Tukey’s multiple comparisons test; data not shown).

### Changes in Gene Expression Relative to Levels of p16 Gene Expression

Cellular senescence is a proposed marker of *biological* rather than *chronological* age (Bhatia-Dey et al., 2016). Since the increased *CDKN2A* expression (using primers amplifying both p16INK4A and p14^ARF^) appeared to be due mostly to increased p16 expression, a marker of chronic senescence (Stein et al., 1999), the results from the qPCR study were grouped into tissues expressing high or low levels of *CDKN2A* about the median value for the region, divided into “high” or “low” *CDKN2A* expression groups. The age of the patient was not considered. Expression levels of the other tested genes were then compared between these groups (Figure 3 and Supplementary Table 3).

More genes showed statistically significant, positive association with the higher levels of *CDKN2A* expression than with age. This occurred with more genes in ascending (16 genes) compared to descending colon (five genes) (Figures 3A,B). *CDKN2A*-associated upregulations were found in ascending colon for one of seven inflammation genes, 0/1 senescence genes, 5/13 oxidative stress genes, 5/6 autophagy genes, 2/6 axonal transport genes and 4/10 apoptosis genes (some genes included in multiple categories). No genes were downregulated with high *CDNK2A* expression in either region.

### CD45 Protein

There was no evidence of microscopic inflammation in any section of colon, based on routine tinctorial staining (H&E). Examination of CD45-positive cells showed no age-related changes in numbers of CD45-positive cells in any region of the muscle in ascending or descending colon from the adult or elderly patient groups (Figure 4 and Supplementary Figure 4). CD45 protein is expressed on many cells of the hematopoietic system, including B cells and T cells (Rheinlander et al., 2018).

### p16 Protein

Given the potentially causative role for senescence in the pathways of aging (see section “Introduction”), immunofluorescence was used to investigate the location of p16 staining within the colon and determine if staining levels change with age.

Immunofluorescence was carried out in sections of full thickness (mucosa removed) ascending and descending colon from the adult and elderly groups using p16, DAPI, and either MAP2 as a neuronal marker, or S100, as a glial cell marker. Very thin 4 μm sections were used to facilitate the localization of staining during imaging and automated quantification. The p16 antibody co-stained with MAP2 (respectively in 8.6% (65 of 755 cells), 26.0% (231/890 cells), 13.3% (272/2,041 cells) and 11.6% (170/1,468 cells) of cells in adult and elderly ascending colon and in adult and elderly descending colon, and 0.0% (0/557 cells) for negative controls, as defined by cells which show staining over 50% of the area of cytoplasm) with little or no co-staining with S100 or DAPI in any of the cells studied, and little or no staining in the smooth muscle (Figure 5). The p16 antibody consistently showed strong and defined co-staining with the MAP2 antibody, often clearly demonstrating the shape of the cell (Figures 5, 6). Controls for the p16 antibody determined reliability; sections of endocervical adenocarcinoma served as a positive control, where staining occurred in both cytoplasm and nucleus (Supplementary Figure 1). Additionally, p16 blocking peptide blocked binding of p16, but not MAP2 antibody. These controls also served to exclude the possibility of lipofuscin autofluorescence as the source of the signal (Supplementary Figure 2).

Cytoplasmic and nuclear p16 staining within the cell bodies of MAP2-positive neurons were analyzed as a percentage of area covered (see section “Materials and Methods”; Figures 6A–C′), and by the average level of fluorescence (see section “Materials and Methods”) in the region of interest. In total, 5,710 neurons from 52 patients were analyzed between the four groups. No age-related changes in p16 protein staining were found in descending colon (Figures 6D–G). In ascending colon, p16 protein staining was increased within the neuronal cell body cytoplasm of the elderly compared to adults. This was detected by measuring the area of p16 coverage and the level of fluorescence in the neuronal cytoplasm (Figures 6D,F). There was no age-related change in area of p16 staining within neuronal nuclei, and although an increase in the level of p16 fluorescence occurred in the elderly compared to the adults (Figures 6E,G), the staining was largely peripheral and not possible to exclude from overlap with the cytoplasmic staining (Supplementary Figure 3).

## Discussion

This study demonstrated increased p16 expression within myenteric neurons of the aged human colon. An initial search for age-related changes in gene expression in the intact colon (mucosa removed but containing multiple cell-types including muscle and enteric neurons) highlighted the possibility of small increases in *CDKN2A* (when using primers for p16^INK4A^ and p14^ARF^ together but not for p14^ARF^ alone) and certain other genes. Notably the latter were more associated with increased expression of *CDKN2A* rather than temporal age. On this basis, p16 immunostaining was performed, to examine the potential for increases in p16^INK4A^ expression and if confirmed, determine the location and level of protein expression.

A hallmark of senescence is the exit of a proliferating cell from the cell cycle. This involves interaction of p16 with CDK4, so p16 is typically considered a nuclear protein (Serrano et al., 1993; Ciesielska et al., 2017). Thus, the second part of the study began with the hypothesis that upregulated *CDKN2A* would be reflected by p16 staining in the nuclei of cells undergoing proliferation within the aged human colon, The focus was on enteric neurons and glial cells (see section “Introduction”). However, S100-positive glial cells exhibited little-or-no p16 staining. Rather, high levels of staining were found in nerve cell bodies of the myenteric plexus, but within the cytoplasm and not clearly within the nucleus. Antibody fluorescence was measured as a representative of protein expression. Although inter-individual variability was high, the data showed that cytoplasmic fluorescence was increased in the neurons of ascending but not descending colon of the elderly, suggesting region-dependent upregulation of p16 during aging within enteric neurons.

Understanding the mechanisms of declining neuromuscular functions could identify ways of promoting healthy aging of the bowel. However, experiments are hampered by lack of routinely available primary cultures of multiple human cell-types. Accordingly, this study investigated the intact colon (mucosa removed) and began by using qPCR to look for age-related expression of genes in degenerative pathways, including cellular senescence, mitochondrial dysfunction and low-grade inflammation (Lopez-Otin et al., 2013; Bhatia-Dey et al., 2016; Franceschi et al., 2017). This approach has several weaknesses, including the limited number of genes examined, together with any unintended selection bias. Further, the use of intact colon means that relatively small amounts of extracted mRNA are likely to be derived from neurons and other cells embedded within the much larger muscle mass. Thus, if genes of interest are in neurons at low copy number, statistically significant increases are difficult to detect, compared with genes which have a high intensity of expression in neurons (e.g., the present study detected *TUBB3*). An additional weakness was the use of primers for *CDKN2A* encoding p16^INK4A^ and p14^ARF^, although this was mitigated to some extent by additional experiments with primers specific for p14^ARF^ alone. Nevertheless, although only an exploratory study, the results suggested an increase in expression of *CDKN2A* (encoding p16^INK4A^ and p14^ARF^, not p14^ARF^ alone) and several other genes within the ascending colon of the elderly, with increased expression of a smaller number in descending colon.

*CDKN2A* (p16^INK4A^ and p14^ARF^, not p14^ARF^ alone) was the only gene tested in which expression was upregulated in both ascending and descending colon from the elderly (although fold change was greater in ascending colon). By contrast, *CDKN1A* (encoding the senescence protein p21), showed no changes with age in either region of colon. Although not an exclusive divide (Krishnamurthy et al., 2004), the latter is often thought to be involved mostly with “acute” senescence at all ages, whereas p16 is important for “chronic” senescence, being associated with aging and a failure of the immune system to clear senescent cells (Stein et al., 1999). Indeed, chronic senescence may be a better marker of how tissue ages in response to genetic and environmental influences over time (Bhatia-Dey et al., 2016). For this reason, the gene expression data from the present study were reanalyzed according to the degree of expression of *CDKN2A*. The results showed that high levels of *CDKN2A* were associated with increased expression of a greater number of genes, compared with the number associated with advanced age, especially in ascending colon. Interestingly, the qPCR data showed some association between *CDKN2A* and components of the SASP, including TNF, VEGFB and genes involved with oxidative stress (expression of IL-6, often associated with SASP, was unchanged). Notably, the SASP may be context-dependent with different profiles, depending on cell type and cause of senescence (Gorgoulis et al., 2019).

To explore a possible association between chronic senescence and the aged colon, an immunofluorescence study was undertaken with p16. As a control to examine the integrity of the tissue, an immunohistochemistry study with CD45 demonstrated an absence of confounding increases in hematopoietic cells in each of the age groups and regions of colon investigated. Although a greater number of samples is now needed to make definitive conclusions, the subsequent p16 immunofluorescence study (*n* = 12–14 each age group and region of colon; *N* = 52 total) showed increased staining in myenteric nerve cell bodies of ascending colon from the elderly. Interestingly, this was not replicated in descending colon, indicating that the age-related increase in *CDKN2A* expression in this region of colon did not translate to increased p16 protein staining; the reasons for this lack of translation are not clear but could be related to the relatively small increase in *CDKN2A* in descending colon.

To investigate the biology of human GI neuromuscular and other functions it is important to use human GI tissues, often removed at surgery for disease. This means that to make progress such studies must confront any influence of the disease on the data generated, in addition to variations in patient age, gender and genetic background (Sanger et al., 2013). As in the present study, the use of an optimal sample size and an understanding of the clinical history of the tissue donors are part of the methods used to minimize variability. Thus, if properly controlled, a strength of the use of human tissues is that this can highlight differences between animal and human GI functions, especially important when conclusions are based on particular strains of rodents with unproved translational significance (Sanger et al., 2013). An investigation into the role of p16 in the aging human bowel presents its own challenges because although all tissues, irrespective of colon region and age, were taken as “macroscopically normal” sections 5–10 cm away from the tumor in cancer patients (see section “Human Tissues”), the possibility remains that the cancer itself could have influenced the expression of p16 and not aging. Thus, p16 may occur in the cytoplasm of carcinoma cells (colorectal, head and neck squamous cell, melanoma, astrocytomas; Straume et al., 2000; Zhao et al., 2003, 2012; Arifin et al., 2006), perhaps to inhibit nuclear function (Nilsson and Landberg, 2006). Nevertheless, the present data are striking because although all tissues were removed from cancer patients the region- and age-dependent increase in p16 staining argues in favor of an association with aging. Such a conclusion finds consistency with a previous report of an age-related decline in cholinergic function in the same ascending colon region, not in the descending colon (Broad et al., 2019).

It has conventionally been thought that senescence occurs in mitotic cells, involving upregulation of cell cycle-related genes (including p16), accompanied by exit from the cell cycle (Carnero, 2013). Nevertheless, Post-Mitotic Cell Senescence (PoMiCS) has been described, in which post-mitotic cells activate p16 and/or the p53/p21 axis, accumulate lipofuscin (“age pigment”; another indicator of senescence; Moreno-García et al., 2018) and secrete components of the SASP (Jurk et al., 2012; Sapieha and Mallette, 2018). This has been found in intestinal myenteric neurons of mice, increasing in frequency during advancing age (Jurk et al., 2012). PoMiCS cells exhibited severe DNA damage and oxidative stress, expressed markers of senescence, including p16, and demonstrated morphological changes typical of senescent cells, e.g., accumulation of lipofuscin, increased size of mitochondria, and reduced heterochromatin (Tan et al., 2014; Wan et al., 2014; Ishikawa and Ishikawa, 2019). Ishikawa and Ishikawa (2019) showed immunofluorescence images demonstrating cytoplasmic p16 protein staining in long-term cultured post-mitotic rat neurons. The present study now suggests that PoMiCS could occur within human myenteric neurons of the elderly.

Staining of the neuronal cytoplasm by the p16 antibody, with little-or-no nuclear staining, was not anticipated since p16 acts as a negative regulator of cell cycle progression at the G1 checkpoint in the nucleus (Serrano et al., 1993). Nevertheless, p16 has been reported to have roles in non-cell cycle-related processes including anoikis, matrix-dependent cell migration, protection from DNA damage and neuroprotection in aging mice (Fahraeus and Lane, 1999; Plath et al., 2000; Sen et al., 2017; Micheli et al., 2019), and in addition, has been shown to bind to cytoplasmic proteins (e.g., cytoplasmic β-actin and γ-actin, and Rin2; Souza-Rodrigues et al., 2007). Further, two forms of p16 are proposed, one in both the nucleus and cytoplasm, the other specific to the cytoplasm: Nilsson and Landberg, 2006). This evidence could suggest cytoplasmic roles for p16 unrelated to the cell cycle.

It is not clear if cytoplasmic p16 protein expression in myenteric neurons is involved with senescence or any of the above-described activities of p16, exerting a pathological or beneficial activity in the aging colon. Confirmatory studies are now needed to determine the presence or absence of other senescence and SASP protein markers in the neurons, and whether such changes are regulated by cytoplasmic p16.

### Conclusion

The present study in human colon supports the wealth of literature which implicates p16 as a key component in aging. In particular, the correlations between age, colon region, and cytoplasmic p16 staining in the myenteric neurons suggests a role for p16 in post-mitotic human enteric neurons during aging. Further experiments are needed to explore this possibility, perhaps by western blotting with human isolated primary cultures of human enteric neurons, and by double-labeling of neurons to identify the nerve phenotype expressing p16. In particular, other markers of senescence (e.g., senescence-associated beta-galactosidase) must be examined to determine if the increase in p16 expression reflects senescence or some other age-related role of p16. Regardless, the data is consistent with the suggestion that ascending colon is an important area in age-related decline of human colon functions, leading to reduced enteric nerve reserve capacity and increased likelihood of developing bowel disorders (Broad et al., 2019). The reason for region-dependence is not understood. Changes in intestinal microbiota during advanced age are reported (Vaiserman et al., 2017) including differences in metabolic and fermentation profiles in different colonic regions (Firrman et al., 2020). For example, concentrations of short chain fatty acids and fermentable substrates are higher in ascending colon, being associated with inflammatory changes (Macfarlane et al., 1992; Karlund et al., 2019). Perhaps these and other substances damage myenteric neurons within the ascending colon, aided by longer storage of food residue in this region compared to descending/sigmoid colon (Proano et al., 1990) and increased mucosal permeability during advancing age (Nicoletti, 2015, but see one large-scale study *in vitro* which found no change in mucosal permeability; Krueger et al., 2016).

## Data Availability Statement

The original contributions presented in the study are included in the article/Supplementary Material, further inquiries can be directed to the corresponding author.

## Ethics Statement

The studies involving human participants were reviewed and the study was approved by the East London Research Ethics Committee (REC: 10/HO703/71) and subsequently by the London City Road and Hampstead Research Ethics Committee (REC: 15/LO/21/27). All patients were fully informed of the aims of the study and use of tissue, but were not involved in study design, interpretation of results, or writing/editing of this report. Written informed consent was obtained from all patients undergoing surgery, for use of macroscopically-normal ascending and descending/sigmoid colon. The patients/participants provided their written informed consent to participate in this study.

## Author Contributions

AP performed the qPCR, the immunohistochemistry and subsequent analysis. SEp, EC, and YX performed immunohistochemistry and subsequent analysis. LG performed immunohistochemistry analysis. AP supervised the immunohistochemistry studies, with interpretation guided by JM. CB provided the p16 antibody and its validation. SEp, AP, EC, SEl, JC-A, and CK were instrumental in obtaining human tissue. ME guided interpretation of qPCR analysis. AP and GS designed the study and wrote the manuscript. GS obtained the funding. All authors had access to the study data, critiqued, read and approved the manuscript.

## Conflict of Interest

GS received funding from Takeda Pharmaceuticals. The funder was not involved in the study design, collection, analysis, interpretation of data, the writing of this article, or the decision to submit it for publication. ME was involved in some parts of the analysis while she was employed by Takeda Pharmaceuticals, United States. The remaining authors declare that the research was conducted in the absence of any commercial or financial relationships that could be construed as a potential conflict of interest.

## Publisher’s Note

All claims expressed in this article are solely those of the authors and do not necessarily represent those of their affiliated organizations, or those of the publisher, the editors and the reviewers. Any product that may be evaluated in this article, or claim that may be made by its manufacturer, is not guaranteed or endorsed by the publisher.
