# Supplementary material for: Expression of p16 Within Myenteric Neurons of the Aged Colon: A Potential Marker of Declining Function
Source: Front Neurosci. 2021 Oct 7;15:747067. doi: 10.3389/fnins.2021.747067 (PMC8529329; doi:10.3389/fnins.2021.747067)

Supplementary Material

**Supplementary Table 1.** Genes studied using qPCR, with molecular pathways they affect and brief descriptions.

| **Gene** | **Protein Name** | **Relevant Pathway(s)** | **Description** |
| --- | --- | --- | --- |
| ***ANO1*** | Anoctamin-1 | Marker for ICCs | Calcium-activated chloride channel |
| ***ATG5*** | Autophagy-related 5 | Autophagy, inflammation, apoptosis | Autophagosome assembly, axon morphology, B and T cell survival and proliferation |
| ***ATG7*** | Autophagy-related 7 | Autophagy, apoptosis | Autophagosome assembly, modulates p53 activity |
| ***CAT*** | Catalase | Oxidative stress | Clearance of ROS |
| ***CDKN1A*** | Cyclin-dependent kinase inhibitor 1A, p21 | Senescence | Cell cycle progression at the G1/S transition |
| ***CDKN2A*** | Cyclin-dependent kinase inhibitor 2A, p16INK4A and p14^ARF^ | Senescence (p16INK4A) and apoptosis (p14^ARF^) | Cell cycle progression at the G1/S transition |
| ***CHAT*** | Choline acetyltransferase | Marker for cholinergic neurons | Synthesises acetylcholine |
| ***DCTN1*** | Dynactin subnunit 1 | Axonal transport | Retrograde motility of vesicles and organelles along microtubules |
| ***DCTN2*** | Dynactin subunit 2, Dynamitin | Axonal transport | Retrograde motility of vesicles and organelles along microtubules |
| ***DUOX2*** | Dual oxidase 2 | Oxidative stress | Generation of ROS |
| ***DYNC1H1*** | Dynein cytoplasmic 1 heavy chain 1 | Axonal transport | Retrograde motility of vesicles and organelles along microtubules |
| ***DYNLL1*** | Dynein light chain 1, cytoplasmic | Axonal transport, apoptosis | Retrograde motility of vesicles and organelles along microtubules, pro-apoptotic activity |
| ***ELN*** | Elastin | Extracellular matrix | Elastic extracellular matrix structural protein |
| ***GPX1*** | Glutathione peroxidase 1 | Oxidative stress | Clearance of ROS |
| ***GPX3*** | Glutathione peroxidase 3 | Oxidative stress | Clearance of ROS |
| ***GPX4*** | Glutathione peroxidase 4 | Oxidative stress | Clearance of ROS |
| ***HSF1*** | Heat shock factor 1 | Apoptosis, oxidative stress | Cellular response to multiple stressors |
| ***HTR4*** | 5-Hydroxytryptamine receptor 4 | Marker for serotonergic neurons | Serotonin receptor |
| ***IL13*** | Interleukin-13 | Inflammation | Cytokine, may be pro- or anti-inflammatory |
| ***IL6*** | Interleukin-6 | Inflammation | Proinflammatory cytokine |
| ***KIF1B*** | Kinesin family member 1B | Axonal transport, apoptosis | Anterograde transport of mitochondria and synaptic vesicles, neuronal apoptosis |
| ***KIT*** | Proto-oncogene c-KIT | Marker for ICCs | Transmembrane receptor for Stem Cell Factor |
| ***KLC1*** | Kinesin light chain 1 | Axonal transport | Coupling of cargo to kinesin molecules for retrograde transport |
| ***MPV17*** | Mitochondrial Inner Membrane Protein MPV17 | Oxidative stress | Metabolism of ROS |
| ***MYH11*** | Myosin-11 | Marker for smooth muscle | Contractile protein in smooth muscle |
| ***NOS1*** | Nitric oxide synthase 1 (Neuronal) | Marker for nitrergic neurons | Synthesises nitric oxide |
| ***NOS2*** | Nitric oxide synthase 2 (inducible) | Marker for nitrergic neurons, inflammation | Synthesises nitric oxide, induced by immune response |
| ***NOX4*** | NADPH oxidase 4 | Oxidative stress, apoptosis | Generation of ROS |
| ***PINK1*** | PTEN-induced kinase 1 | Autophagy, oxidative stress | Clearance of damaged mitochondria |
| ***PRDX1*** | Peroxiredoxin-1 | Oxidative stress | Clearance of ROS |
| ***PRKCD*** | Protein kinase C δ-type | Oxidative stress, apoptosis | Calcium-independent protein kinase, can be pro- or anti-apoptotic depending on context, involved in ROS production |
| ***PRKN*** | Parkin | Autophagy, oxidative stress | Clearance of damaged mitochondria |
| ***S100B*** | S100 calcium binding protein B | Marker for glial cells, inflammation | Secreted in response to immune system or neurotransmitters, may have role in cell cycle progression and neurite extension |
| ***SOD1*** | Superoxide dismutase 1 | Oxidative stress | Clearance of ROS |
| ***SOD2*** | Superoxide dismutase 2 | Oxidative stress | Clearance of ROS |
| ***TBK1*** | TANK-binding kinase 1 | Inflammation | Protein kinase with anti-inflammatory activity |
| ***TIE1*** | Tyrosine kinase with immunoglobulin-like and EGF-like domains 1 | Angiogenesis | Regulation of angiogenesis |
| ***TNF*** | Tumour necrosis factor α | Inflammation, apoptosis | Proinflammatory cytokine |
| ***TUBB3*** | βIII-Tubulin | Marker for neurons | Heterodimerises with α-tubulins and polymerises to form microtubules |
| ***VCP*** | Valosin-containing protein, p97 | Autophagy | Protein degradation, intracellular membrane fusion, DNA repair and replication, cell cycle regulation |
| ***VEGFA*** | Vascular endothelial growth factor A | Angiogenesis, apoptosis | Induces angiogenesis, anti-apoptotic |
| ***VEGFB*** | Vascular endothelial growth factor B | Angiogenesis | Induces angiogenesis |
| ***WDR45*** | WD repeat domain 45 | Autophagy | Autophagosome assembly |

**Supplementary Table 2.** Relative expression levels of genes between colon regions and age, either adult or elderly, normalised to adult ascending tissue. P values calculated using the student’s *t*-test to compare between age groups within a region. Statistically significant (<0.05) P values shown in bold.

|  | **Ascending** | | | **Descending** | | |
| --- | --- | --- | --- | --- | --- | --- |
|  | **Average RQ ± Standard Error** | | **P-value** | **Average RQ ± Standard Error** | | **P-value** |
| **Gene** | **Adult** | **Elderly** |  | **Adult** | **Elderly** |  |
| ***ANO1*** | 1.00 ± 0.16 | 0.98 ± 0.12 | 0.93 | 0.76 ± 0.11 | 0.73 ± 0.07 | 0.82 |
| ***ATG5*** | 1.00 ± 0.20 | 1.17 ± 0.24 | 0.10 | 1.00 ± 0.16 | 0.90 ± 0.20 | 0.24 |
| ***ATG7*** | 1.00 ± 0.28 | 1.12 ± 0.28 | 0.38 | 0.83 ± 0.16 | 0.98 ± 0.25 | 0.13 |
| ***CAT*** | 1.00 ± 0.09 | 1.28 ± 0.14 | 0.11 | 0.97 ± 0.31 | 1.01 ± 0.32 | 0.79 |
| ***CDKN1A*** | 1.00 ± 0.25 | 0.63 ± 0.12 | 0.19 | 0.73 ± 0.20 | 1.02 ± 0.37 | 0.48 |
| ***CDKN2A*** (***p14^ARF^)*** | 1.00 ± 0.21 | 0.58 ± 0.39 | 0.07 | 0.69 ± 0.35 | 0.86 ± 0.31 | 0.43 |
| ***CDKN2A*** (***p14^ARF^ and p16^INK4A^)*** | 1.00 ± 0.19 | 1.62 ± 0.21 | **0.04** | 0.89 ± 0.08 | 1.46 ± 0.21 | **0.02** |
| ***CHAT*** | 1.00 ± 0.15 | 0.83 ± 0.11 | 0.37 | 0.90 ± 0.08 | 0.83 ± 0.12 | 0.63 |
| ***DCTN1*** | 1.00 ± 0.36 | 1.14 ± 0.45 | 0.46 | 0.79 ± 0.18 | 0.83 ± 0.17 | 0.58 |
| ***DCTN2*** | 1.00 ± 0.22 | 1.16 ± 0.27 | 0.16 | 0.86 ± 0.15 | 0.87 ± 0.18 | 0.93 |
| ***DUOX2*** | 1.00 ± 0.15 | 1.19 ± 0.45 | 0.71 | 0.87 ± 0.18 | 0.76 ± 0.19 | 0.67 |
| ***DYNC1H1*** | 1.00 ± 0.23 | 1.15 ± 0.17 | 0.13 | 1.02 ± 0.18 | 1.00 ± 0.13 | 0.78 |
| ***DYNLL1*** | 1.00 ± 0.15 | 1.17 ± 0.23 | 0.07 | 0.91 ± 0.23 | 0.87 ± 0.09 | 0.65 |
| ***ELN*** | 1.00 ± 0.52 | 0.95 ± 0.42 | 0.80 | 0.73 ± 0.55 | 0.51 ± 0.27 | 0.27 |
| ***GPX1*** | 1.00 ± 0.09 | 1.23 ± 0.11 | 0.12 | 1.10 ± 0.33 | 0.95 ± 0.15 | 0.23 |
| ***GPX3*** | 1.00 ± 0.12 | 1.57 ± 0.27 | 0.08 | 0.94 ± 0.28 | 1.00 ± 0.36 | 0.66 |
| ***GPX4*** | 1.00 ± 0.05 | 1.34 ± 0.13 | **0.03** | 1.03 ± 0.26 | 0.96 ± 0.17 | 0.47 |
| ***HSF1*** | 1.00 ± 0.26 | 1.22 ± 0.29 | 0.10 | 0.92 ± 0.25 | 0.82 ± 0.15 | 0.27 |
| ***HTR4*** | 1.00 ± 0.12 | 1.19 ± 0.21 | 0.47 | 1.54 ± 0.19 | 1.29 ± 0.19 | 0.36 |
| ***IL13*** | 1.00 ± 0.32 | 0.48 ± 0.10 | 0.12 | 0.54 ± 0.52 | 1.75 ± 3.08 | 0.24 |
| ***IL6*** | 1.00 ± 0.32 | 0.49 ± 0.11 | 0.13 | 0.41 ± 0.12 | 0.78 ± 0.39 | 0.37 |
| ***KIF1B*** | 1.00 ± 0.18 | 1.22 ± 0.20 | **0.03** | 0.98 ± 0.24 | 1.04 ± 0.21 | 0.23 |
| ***KIT*** | 1.00 ± 0.11 | 0.95 ± 0.11 | 0.76 | 0.73 ± 0.07 | 0.84 ± 0.11 | 0.39 |
| ***KLC1*** | 1.00 ± 0.30 | 1.10 ± 0.22 | 0.41 | 0.79 ± 0.19 | 0.83 ± 0.23 | 0.72 |
| ***MPV17*** | 1.00 ± 0.06 | 0.93 ± 0.06 | 0.44 | 0.99 ± 0.25 | 0.90 ± 0.11 | 0.30 |
| ***MYH11*** | 1.00 ± 0.09 | 1.25 ± 0.20 | 0.29 | 1.00 ± 0.05 | 1.01 ± 0.08 | 0.91 |
| ***NOS1*** | 1.00 ± 0.47 | 0.75 ± 0.23 | 0.15 | 0.91 ± 0.34 | 0.94 ± 0.54 | 0.87 |
| ***NOS2*** | 1.00 ± 0.53 | 0.81 ± 0.46 | 0.40 | 1.14 ± 1.58 | 0.67 ± 0.34 | 0.37 |
| ***NOX4*** | 1.00 ± 0.16 | 1.21 ± 0.22 | 0.46 | 0.59 ± 0.07 | 0.95 ± 0.12 | **0.01** |
| ***PINK1*** | 1.00 ± 0.54 | 1.12 ± 0.34 | 0.57 | 0.87 ± 0.20 | 0.75 ± 0.16 | 0.16 |
| ***PRDX1*** | 1.00 ± 0.21 | 1.24 ± 0.26 | **0.04** | 1.01 ± 0.18 | 0.92 ± 0.24 | 0.36 |
| ***PRKCD*** | 1.00 ± 0.08 | 1.16 ± 0.12 | 0.31 | 1.04 ± 0.17 | 0.88 ± 0.08 | 0.40 |
| ***PRKN*** | 1.00 ± 0.30 | 1.22 ± 0.34 | 0.16 | 1.02 ± 0.29 | 1.01 ± 0.23 | 0.92 |
| ***S100B*** | 1.00 ± 0.12 | 1.03 ± 0.10 | 0.84 | 1.06 ± 0.31 | 1.01 ± 0.39 | 0.72 |
| ***SOD1*** | 1.00 ± 0.11 | 1.22 ± 0.10 | 0.14 | 1.05 ± 0.09 | 0.95 ± 0.13 | 0.53 |
| ***SOD2*** | 1.00 ± 0.11 | 0.99 ± 0.09 | 0.95 | 1.15 ± 0.28 | 1.02 ± 0.12 | 0.67 |
| ***TBK1*** | 1.00 ± 0.19 | 1.11 ± 0.30 | 0.34 | 0.96 ± 0.37 | 1.05 ± 0.16 | 0.48 |
| ***TIE1*** | 1.00 ± 0.39 | 1.23 ± 0.58 | 0.32 | 0.63 ± 0.24 | 0.79 ± 0.25 | 0.15 |
| ***TNF*** | 1.00 ± 0.35 | 2.28 ± 1.46 | **0.02** | 1.14 ± 0.40 | 1.21 ± 0.56 | 0.73 |
| ***TUBB3*** | 1.00 ± 0.29 | 1.00 ± 0.34 | 0.98 | 0.86 ± 0.32 | 0.77 ± 0.35 | 0.57 |
| ***VCP*** | 1.00 ± 0.24 | 1.15 ± 0.19 | 0.16 | 0.91 ± 0.17 | 0.95 ± 0.15 | 0.61 |
| ***VEGFA*** | 1.00 ± 0.34 | 1.15 ± 0.52 | 0.48 | 0.74 ± 0.19 | 1.02 ± 0.76 | 0.28 |
| ***VEGFB*** | 1.00 ± 0.26 | 1.21 ± 0.38 | 0.19 | 0.96 ± 0.26 | 0.87 ± 0.17 | 0.40 |
| ***WDR45*** | 1.00 ± 0.35 | 1.04 ± 0.28 | 0.78 | 0.98 ± 0.32 | 0.84 ± 0.22 | 0.28 |

**Supplementary table 3.** Relative expression levels of genes between colon regions and level of p16 (*CDKN2A*) expression, normalised to ascending tissue with low p16 expression. Groups were divided around the median p16 expression level for each region. P values calculated using the student’s *t-*test to compare between age groups within a region. Statistically significant (<0.05) P values shown in bold.

| **Gene** | **Ascending** | | | **Descending** | | |
| --- | --- | --- | --- | --- | --- | --- |
|  | **Average RQ ± Standard Error** | | **P-value** | **Average RQ ± Standard Error** | | **P-value** |
|  | **High *CDKN2A* expression** | **Low *CDKN2A* Expression** |  | **High *CDKN2A* expression** | **Low *CDKN2A* Expression** |  |
| ***ANO1*** | 1.00 ± 0.12 | 1.43 ± 0.15 | **0.04** | 0.89 ± 0.12 | 0.97 ± 0.09 | 0.61 |
| ***ATG5*** | 1.00 ± 0.21 | 1.22 ± 0.22 | **0.04** | 0.94 ± 0.19 | 0.99 ± 0.19 | 0.56 |
| ***ATG7*** | 1.00 ± 0.19 | 1.29 ± 0.33 | **0.03** | 0.84 ± 0.12 | 1.13 ± 0.24 | **0.003** |
| ***CAT*** | 1.00 ± 0.11 | 1.21 ± 0.12 | 0.23 | 0.85 ± 0.21 | 1.07 ± 0.34 | 0.11 |
| ***CDKN2A (p14^ARF^)*** | 1.00 ± 0.35 | 0.73 ± 0.45 | 0.32 | 0.87 ± 0.37 | 0.83 ± 0.38 | 0.87 |
| ***CDKN1A*** (***p14^ARF^ and p16^INK4A^)*** | 1.00 ± 0.28 | 1.11 ± 0.25 | 0.76 | 1.25 ± 0.49 | 1.06 ± 0.26 | 0.74 |
| ***CHAT*** | 1.00 ± 0.21 | 1.35 ± 0.10 | 0.14 | 1.17 ± 0.11 | 1.07 ± 0.15 | 0.60 |
| ***DCTN1*** | 1.00 ± 0.35 | 1.30 ± 0.48 | 0.14 | 0.80 ± 0.21 | 0.94 ± 0.14 | 0.09 |
| ***DCTN2*** | 1.00 ± 0.22 | 1.19 ± 0.26 | 0.10 | 0.84 ± 0.17 | 0.91 ± 0.16 | 0.35 |
| ***DUOX2*** | 1.00 ± 0.44 | 0.98 ± 0.16 | 0.96 | 0.81 ± 0.19 | 0.66 ± 0.14 | 0.55 |
| ***DYNC1H1*** | 1.00 ± 0.23 | 1.19 ± 0.16 | 0.05 | 0.97 ± 0.17 | 1.08 ± 0.13 | 0.11 |
| ***DYNLL1*** | 1.00 ± 0.13 | 1.21 ± 0.22 | **0.03** | 0.90 ± 0.22 | 0.91 ± 0.11 | 0.87 |
| ***ELN*** | 1.00 ± 0.47 | 1.29 ± 0.58 | 0.26 | 0.90 ± 0.64 | 0.57 ± 0.32 | 0.17 |
| ***GPX1*** | 1.00 ± 0.09 | 1.36 ± 0.10 | **0.02** | 1.06 ± 0.28 | 1.11 ± 0.29 | 0.68 |
| ***GPX3*** | 1.00 ± 0.16 | 1.82 ± 0.25 | **0.02** | 0.86 ± 0.29 | 1.27 ± 0.27 | **0.005** |
| ***GPX4*** | 1.00 ± 0.11 | 1.13 ± 0.10 | 0.38 | 0.87 ± 0.15 | 0.93 ± 0.24 | 0.50 |
| ***HSF1*** | 1.00 ± 0.29 | 1.33 ± 0.24 | **0.02** | 0.87 ± 0.27 | 0.96 ± 0.15 | 0.38 |
| ***HTR4*** | 1.00 ± 0.13 | 1.51 ± 0.23 | 0.08 | 1.45 ± 0.24 | 1.82 ± 0.18 | 0.23 |
| ***IL13*** | 1.00 ± 0.24 | 0.99 ± 0.39 | 0.98 | 2.12 ± 3.84 | 1.36 ± 2.27 | 0.60 |
| ***IL6*** | 1.00 ± 0.32 | 0.69 ± 0.23 | 0.42 | 0.74 ± 0.42 | 0.62 ± 0.22 | 0.79 |
| ***KIF1B*** | 1.00 ± 0.20 | 1.18 ± 0.21 | 0.07 | 0.88 ± 0.23 | 1.04 ± 0.19 | 0.12 |
| ***KIT*** | 1.00 ± 0.12 | 1.12 ± 0.12 | 0.49 | 0.75 ± 0.07 | 0.96 ± 0.11 | 0.13 |
| ***KLC1*** | 1.00 ± 0.23 | 1.31 ± 0.25 | **0.01** | 0.80 ± 0.21 | 0.99 ± 0.21 | 0.06 |
| ***MPV17*** | 1.00 ± 0.05 | 1.12 ± 0.08 | 0.20 | 0.98 ± 0.20 | 1.10 ± 0.22 | 0.22 |
| ***MYH11*** | 1.00 ± 0.10 | 1.09 ± 0.18 | 0.67 | 0.84 ± 0.06 | 1.02 ± 0.06 | **0.04** |
| ***NOS1*** | 1.00 ± 0.50 | 1.17 ± 0.46 | 0.45 | 1.04 ± 0.49 | 1.28 ± 0.61 | 0.34 |
| ***NOS2*** | 1.00 ± 0.40 | 0.72 ± 0.50 | 0.20 | 1.17 ± 1.49 | 0.55 ± 0.26 | 0.21 |
| ***NOX4*** | 1.00 ± 0.14 | 1.72 ± 0.26 | **0.03** | 0.73 ± 0.08 | 1.19 ± 0.14 | **0.01** |
| ***PINK1*** | 1.00 ± 0.29 | 1.52 ± 0.58 | **0.03** | 0.96 ± 0.30 | 0.98 ± 0.11 | 0.84 |
| ***PRDX1*** | 1.00 ± 0.05 | 1.40 ± 0.08 | **0.0005** | 0.98 ± 0.15 | 1.09 ± 0.28 | 0.32 |
| ***PRKCD*** | 1.00 ± 0.12 | 1.13 ± 0.10 | 0.39 | 1.05 ± 0.17 | 0.84 ± 0.07 | 0.26 |
| ***PRKN*** | 1.00 ± 0.33 | 1.23 ± 0.32 | 0.14 | 0.98 ± 0.31 | 1.06 ± 0.21 | 0.49 |
| ***S100B*** | 1.00 ± 0.13 | 1.32 ± 0.10 | 0.07 | 1.13 ± 0.40 | 1.25 ± 0.39 | 0.51 |
| ***SOD1*** | 1.00 ± 0.09 | 1.26 ± 0.11 | 0.09 | 0.93 ± 0.08 | 1.11 ± 0.13 | 0.26 |
| ***SOD2*** | 1.00 ± 0.09 | 1.12 ± 0.12 | 0.43 | 0.97 ± 0.14 | 1.34 ± 0.29 | 0.26 |
| ***TBK1*** | 1.00 ± 0.17 | 1.19 ± 0.30 | 0.11 | 0.89 ± 0.24 | 1.19 ± 0.28 | **0.02** |
| ***TIE1*** | 1.00 ± 0.33 | 1.69 ± 0.61 | **0.008** | 0.74 ± 0.22 | 0.98 ± 0.35 | 0.09 |
| ***TNF*** | 1.00 ± 0.45 | 1.93 ± 1.35 | 0.07 | 1.10 ± 0.57 | 1.15 ± 0.33 | 0.82 |
| ***TUBB3*** | 1.00 ± 0.29 | 1.51 ± 0.32 | **0.002** | 1.10 ± 0.43 | 0.98 ± 0.42 | 0.53 |
| ***VCP*** | 1.00 ± 0.10 | 1.32 ± 0.24 | **0.002** | 0.95 ± 0.18 | 1.06 ± 0.16 | 0.18 |
| ***VEGFA*** | 1.00 ± 0.25 | 1.38 ± 0.59 | 0.09 | 1.09 ± 0.77 | 0.87 ± 0.45 | 0.45 |
| ***VEGFB*** | 1.00 ± 0.22 | 1.36 ± 0.39 | **0.03** | 0.98 ± 0.30 | 0.97 ± 0.17 | 0.95 |
| ***WDR45*** | 1.00 ± 0.27 | 1.38 ± 0.35 | **0.02** | 1.06 ± 0.39 | 1.07 ± 0.25 | 0.94 |

**Supplementary Table 4.** Primers used for qPCR.

| **Gene** | **Forward Primer (5’ to 3’)** | **Reverse Primer (5’ to 3’)** | **Ampli-con Size (bp)** |
| --- | --- | --- | --- |
| ***ANO1*** | AATCCACGGAGTCGGGTTTG | TTTGGGCTGGATGGGATCTG | 178 |
| ***ATG5*** | TGCAGATGGACAGTTGCACA | CCACTGCAGAGGTGTTTCCA | 139 |
| ***ATG7*** | TCCTTTTGGAACAAGCAGCAAA | AAGACAGAGGGCAGGATAGC | 158 |
| ***ATP5B*** | Proprietary (Primerdesign) | Proprietary (Primerdesign) | 150 |
| ***CAT*** | AAGACTCCCATCGCAGTTCG | ATTTCACTGCAAACCCACGAG | 91 |
| ***CDKN1A*** | TGGAGACTCTCAGGGTCGAAA | GGCGTTTGGAGTGGTAGAAATC | 65 |
| ***CDKN2A for p14^ARF^*** | GAGAACATGGTGCGCAGGT | GATGTGAACCACGAAAACCCTC | 87 |
| ***CDKN2A for p16^INK4A^ and p14^ARF^*** | CATAGATGCCGCGGAAGGT | CTAAGTTTCCCGAGGTTTCTCAGA | 79 |
| ***CHAT*** | AGCAACCGGTTTGTCCTCTC | TTGTAGCAGGCACCATACCC | 101 |
| ***DCTN1*** | AAGGAGGCGAGAAAGGAAGC | CGCTCTTCAGCCATCTCCTT | 122 |
| ***DCTN2*** | TGGAGACTGTAGAGCTGTTGC | TAGCCGAGCCTCCACTTGA | 77 |
| ***DUOX2*** | CCGGCAATCATCTATGGAGGT | CCTTGGGGCCTCTGGAATT | 125 |
| ***DYNC1H1*** | CAGCAAGCCAACATCCAGTTC | CAAAGGTCATCAGCAGGCAAG | 110 |
| ***DYNLL1*** | TCAGGCGCTGGAGAAATACA | CGAAGTTCCTCCCCACGATG | 110 |
| ***ELN*** | GCTCCTGCTGTCCATCCTC | CCTCCGGGAACTGGCTTA | 162 |
| ***GAPDH^1^*** | CTCTGCTCCTCCTGTTCGAC | TTAAAAGCAGCCCTGGTGAC | 144 |
| ***GPX1*** | TTTGGGCATCAGGAGAACGC | AGCATGAAGTTGGGCTCGAA | 92 |
| ***GPX3*** | CTGACGGGCCAGTACATTGA | AAGCCCAGAATGACCAGACC | 74 |
| ***GPX4*** | CCTCATCGACAAGAACGGCT | AGAAATAGTGGGGCAGGTCC | 89 |
| ***HSF1*** | CCTGGCCATGAAGCATGAGA | GATCCGGTTTGACTGCACCA | 133 |
| ***HTR4*** | CGTTAATGCTGGGAGGCT | ATGGCGTAGGGCTTGTTG | 166 |
| ***IL13*** | CAGAGGATGCTGAGCGGATT | ACTGGGCCACCTCGATTTTG | 94 |
| ***IL6^2^*** | AGCCCTGAGAAAGGAGACATGTA | TCTGCCAGTGCCTCTTTGC | 66 |
| ***KIF1B*** | TGGTCTCATACCTCACCCGAA | AGGCGTGTAAGAGCATTTCCT | 88 |
| ***KIT*** | ACCAACACCGGCAAATACAC | ACCAGCGTGTCGTTGTCTTC | 134 |
| ***KLC1*** | CGTGCACATGAAAGGGAGTT | CTTTGTACCAGCCGCCATAC | 136 |
| ***MPV17*** | GCTTTGCCCCGTGTTTTCTA | AACTGCACAGCAGGCCATAG | 145 |
| ***MYH11*** | CCAAGAACAGGCTTCAGCAG | GTCCCTCTCATCCGCGTATTT | 155 |
| ***NOS1^3^*** | GAGCCAGACAAACCAAAGAAGTAC | GCGCTGGATGGCTTTGAG | 150 |
| ***NOS2^3^*** | CATTCAGATCCCCAAGCTCTACA | TGCCGAGATTTGAGCCTCAT | 150 |
| ***NOX4*** | AAAACCGGCAGAGTTTACCCA | TTTCGGCACAGTACAGGCAC | 125 |
| ***PINK1*** | TCTTTCTGGCCTTCGGGCTA | CAAGCGTCTCGTGTCCAAC | 146 |
| ***PRDX1*** | ACTGGGACCCATGAACATTCC | AAAAGGCCCCTGAACGAGAT | 105 |
| ***PRKCD*** | CTGAGTTCTGGCTGGACCTG | CCTCACTGCGCATAGACTGT | 99 |
| ***PRKN*** | AGCCTGACCAGAGGAAAGTC | GCACTCCCCTTCATGGTACG | 115 |
| ***S100B*** | AGGGAGACAAGCACAAGCTG | GTCCACAACCTCCTGCTCTT | 101 |
| ***SOD1*** | AAAGATGGTGTGGCCGATGT | CAAGCCAAACGACTTCCAGC | 167 |
| ***SOD2^4^*** | TAGCTCTTCAGCCTGCACTG | CTTGTCAAAGGAACCAAAGTCAC | 146 |
| ***TBK1*** | TCCAGTGGATGTTCAAATGAGAGA | CTCCCACATGGACAAAATTCC | 138 |
| ***TIE1*** | CCTGCTTCCAGACAAGGTCA | ATGCCAGTCCAGGGTGTAGA | 136 |
| ***TNF^5^*** | GTGCTTGTTCCTCAGCCTCT | GCTTGTCACTCGGGGTTC | 176 |
| ***TUBB3*** | TGATGAGCATGGCATCGACC | CGAGGCACGTACTTGTGAGA | 111 |
| ***VCP*** | CGCCTAGGGGATGTCATCAG | GTGTCATCAATGGGCAGCAC | 83 |
| ***VEGFA*** | CTTGGGTGCATTGGAGCCTT | CACCAGGGTCTCGATTGGAT | 161 |
| ***VEGFB*** | CAAGTCCGGATGCAGATCCTC | GCTGTGTTCTTCCAGGGACAT | 75 |
| ***WDR45*** | CTACAACGTGGAGCCCTTGAT | CTACTACCACCGCCCACCAA | 120 |

1. Hashemi, A., Roohvand, F., Ghahremani, M.H. (2012). Selection of valid reference genes for expression studies of hepatic cell lines under IFN-alpha treatment. *Biochem. Biophys. Res. Commun.* 426, 649-653.
2. Hubackova, S., Krejcikova, K., Bartek, J., *et al*. (2012). Interleukin 6 signaling regulates promyelocytic leukemia protein gene expression in human normal and cancer cells. *J. Biol. Chem.* 287, 26702-26714.
3. Yip, K.H., Huang, Y., Waye, M.M., *et al*. (2008). Induction of nitric oxide synthases in primary human cultured mast cells by IgE and proinflammatory cytokines. *Int. Immunopharmacol.* 8, 764-768.
4. Zhu, X., Zheng, X., Wu, Y. (2014). Cleaved high molecular weight kininogen stimulates JNK/FOXO4/MnSOD pathway for induction of endothelial progenitor cell senescence*. Biochem. Biophys. Res. Commun.* 450, 1261-1265.
5. Quandt, D., Jasinski-Bergner, S., Muller, U., *et al*. (2014). Synergistic effects of IL-4 and TNFalpha on the induction of B7-H1 in renal cell carcinoma cells inhibiting allogeneic T cell proliferation. *J. Transl. Med.* 12, 151.

**Supplementary figure 1.** p16 antibody detects p16 protein in both cytoplasm and nuclei of control cells. A-A’ and B-B’ demonstrate the use of p16-positive endocervical adenocarcinoma as a control tissue. Primary antibody was not used in A-A’ as a negative control, but was used in B-B’. C-C’ demonstrates p16 expression in a p16-positive breast cancer cell line (MDA-MB-468). Scale bar is 25 µm.


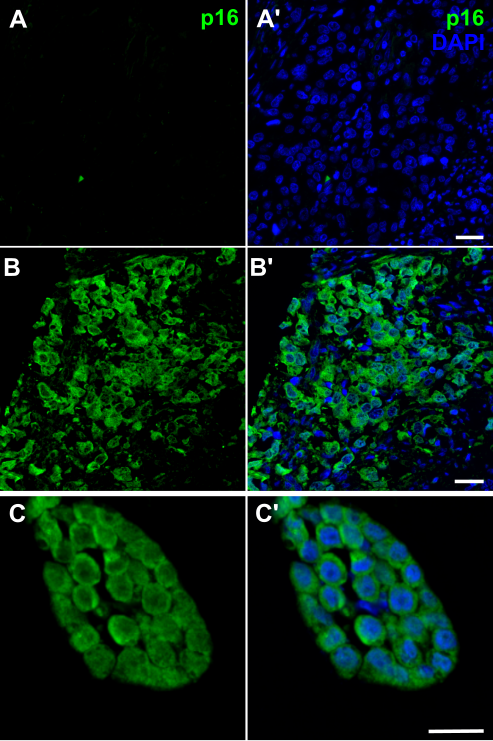


**Supplementary figure 2.** Controls carried out to test p16 antibody. The position of the myenteric plexus is represented by the dotted line. Blocking peptide is against p16 antibody, and in all cases shown has been incubated for one hour at room temperature with the primary antibody prior to use. Successful p16 antibody binding is shown in C-C’’. The antibody demonstrates no fluorescence in the absence of secondary antibody (B-B’’) or after incubation with blocking peptide (D-D’’). Blocking peptide has no effect on the binding of MAP2 antibody (E-E’’ without blocking peptide, F-F’’ with blocking peptide), demonstrating specificity of binding. The absence of fluorescence in the myenteric plexus when no antibody is present (A-A’’) and when the antibody has been pre-treated with blocking peptide (D-D’’) excludes the possibility of signal being due to lipofuscin autofluorescence. Scale bar represents 50 µm.


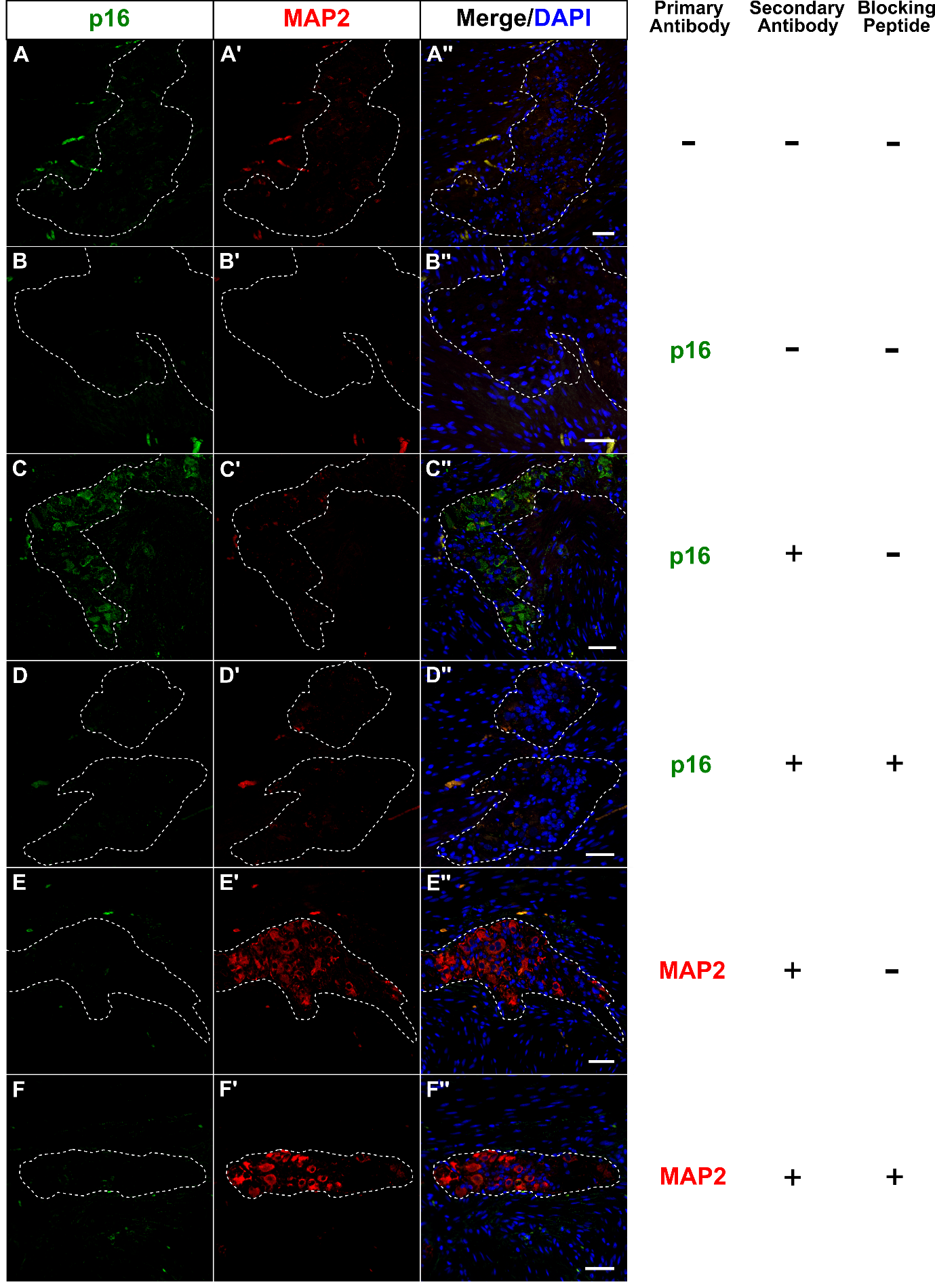


**Supplementary figure 3.** p16 immunofluorescence in neuronal nuclei is often located at the periphery, and may be due to overlap between nucleus and cytoplasm. Individual immunostainings are shown in A-C. DAPI can be seen to overlap with p16 staining in the two cells indicated by arrowheads (D). In the cell indicated by the white arrowhead, this is clearly in a region of overlap between the neuronal cytoplasm, shown by MAP2 staining, and the nucleus, shown by DAPI (E). At the point indicated by the yellow arrowhead, a single focus overlays the nucleus at the extreme periphery (D), although this point also appears to overlap with the MAP2 staining of the cytoplasm (F). Both of these cells would be indicated as having nuclear p16 in the analysis protocol. This peripheral pattern of staining was commonly seen in cells identified as containing nuclear p16, although it is impossible to judge whether this type of staining is cytoplasmic or nuclear using this method. Little-to-no p16 fluorescence was identified in regions of the nucleus not overlapping with MAP2 staining. Scale bar measures 10 µm.


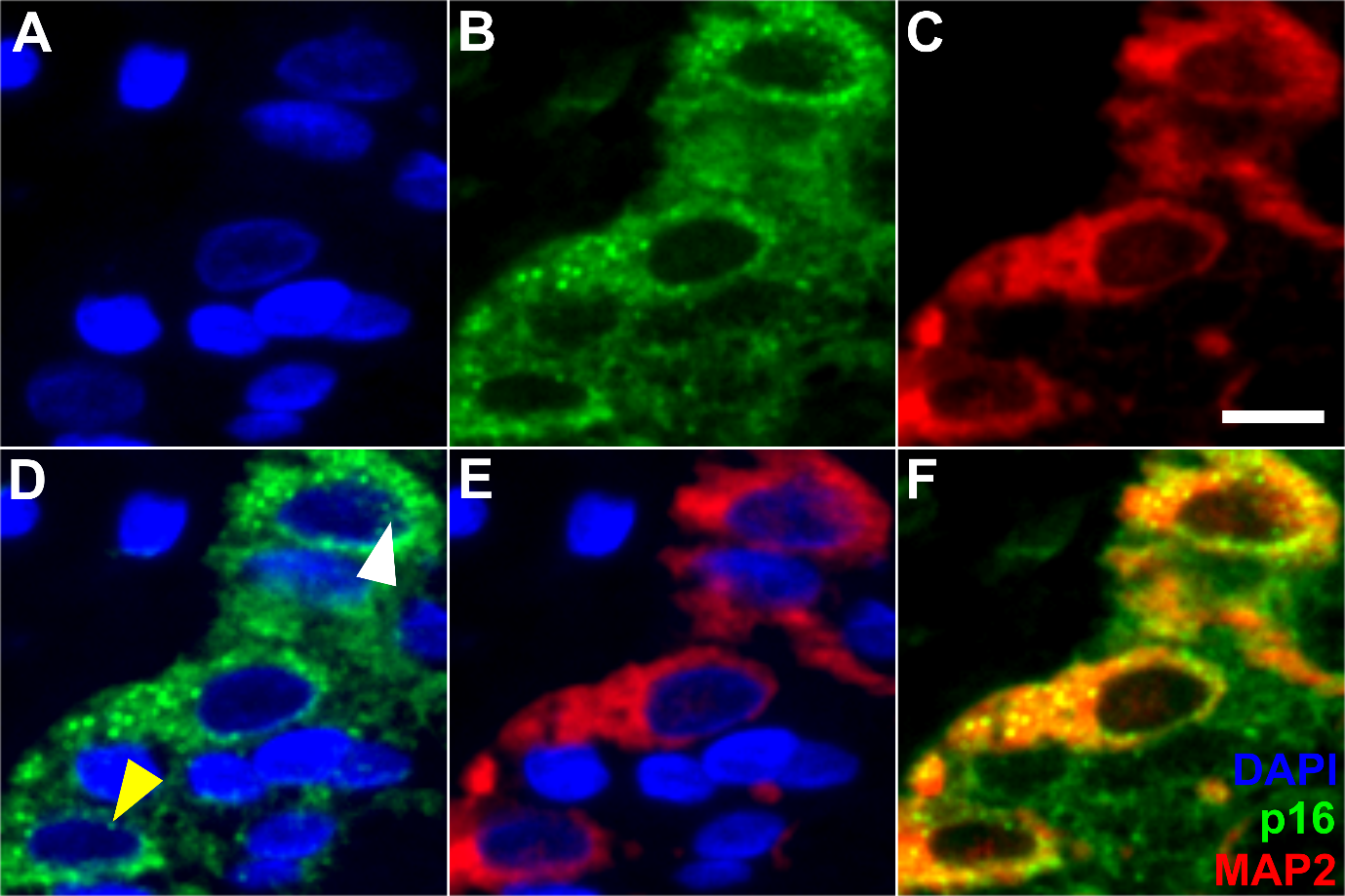


**Supplementary figure 4.** No significant age-related differences were seen in the percentage of CD45-positive cells in colon sections in either ascending or descending colon. Statistical tests compared results for each colon region between age groups; adult (25-60 years old; ●) and elderly (70+ years old; ▲). Bars indicate data mean. Data was analysed by two independent observers, results shown separately in A and B. N values differ slightly between observers due to individual differences in defining whether the samples were able to meet acceptance criteria.


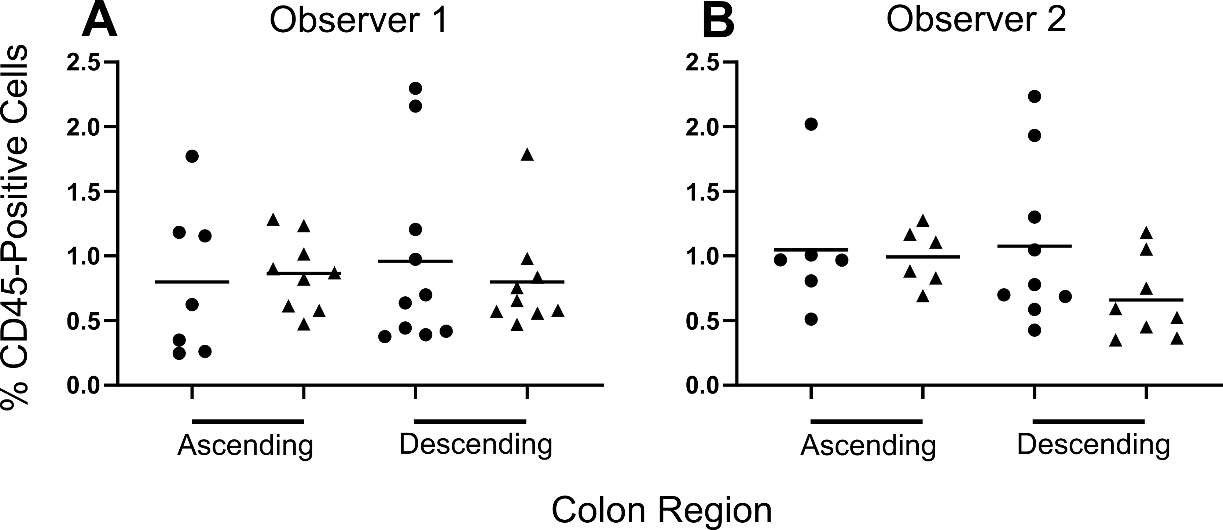

Supplement: Supplementary file 1 [file Data_Sheet_1.docx]
